# LRIG1 controls proliferation of adult neural stem cells by facilitating TGF*β* and BMP signalling pathways

**DOI:** 10.1038/s42003-024-06524-8

**Published:** 2024-07-10

**Authors:** Stephanie Ouzikov, Kyshona M. Edwards, Tanvi Anandampillai, Samuel Watanabe, Daniela Lozano Casasbuenas, Karen K. Siu, Danyon Harkins, Aaron Dou, Danielle Jeong, Jeffrey E. Lee, Scott A. Yuzwa

**Affiliations:** 1 Department of Laboratory Medicine and Pathobiology, Temerty Faculty of Medicine, University of Toronto, 1 King’s College Circle, Toronto, ON M5S 1A8 Canada; 2 Institute of Medical Science, University of Toronto, 1 King’s College Circle, Toronto, ON M5S 1A8 Canada; 3 Program in Neurosciences and Mental Health, Hospital for Sick Children, 686 Bay Street, Toronto, ON M5G 0A4 Canada

**Keywords:** Adult neurogenesis, Molecular neuroscience

## Abstract

Adult Neural Stem Cells (aNSCs) in the ventricular-subventricular zone (V-SVZ) are largely quiescent. Here, we characterize the mechanism underlying the functional role of a cell-signalling inhibitory protein, LRIG1, in the control of aNSCs proliferation. Using *Lrig1* knockout models, we show that *Lrig1* ablation results in increased aNSCs proliferation with no change in neuronal progeny and that this hyperproliferation likely does not result solely from activation of the epidermal growth factor receptor (EGFR). Loss of LRIG1, however, also leads to impaired activation of transforming growth factor beta (TGF*β*) and bone morphogenic protein (BMP) signalling. Biochemically, we show that LRIG1 binds TGF*β*/BMP receptors and the TGF*β*1 ligand. Finally, we show that the consequences of these interactions are to facilitate SMAD phosphorylation. Collectively, these data suggest that unlike in embryonic NSCs where EGFR may be the primary mechanism of action, in aNSCs, LRIG1 and TGF*β* pathways function together to fulfill their inhibitory roles.

## Abstract (web summary)

LRIG1 is an important negative regulator of cell signaling. In this study, loss of LRIG1 leads to hyperproliferation of adult neural stem cells by facilitation of TGF*β* and BMP signalling pathways.

## Introduction

Adult neural stem cell (aNSC) populations reside in two specialized niches in the adult mammalian brain. One of these two aNSCs populations is found lining the lateral ventricles (LV) in a region referred to as the ventricular-subventricular zone (V-SVZ). aNSCs found within this V-SVZ niche, in mice, are largely quiescent in nature, dividing infrequently for olfactory bulb (OB) maintenance and olfactory learning^1^. aNSCs can also be activated to proliferate following injury, producing progeny which migrate to the injury site to repair damage^2–4^. Once activated under homeostatic conditions, aNSCs divide to give rise to transit-amplifying progenitors (TAPs, also called type C cells), that can proliferate and give rise to a new-born neuroblasts (also called type A cells). Neuroblasts migrate to the olfactory bulb to give rise to interneurons that integrate into existing circuits^5^. How quiescent aNSCs are maintained in this state by the complex integration of extrinsic niche signals and intrinsic genetic programs is not fully understood.

Prior work has suggested that aNSCs arise developmentally between embryonic day (E) E13.5 and E15.5 when a population of radial glial progenitors (RGPs) lining the LV transition from an actively proliferating state to a ‘slow-dividing’ state. In so doing, these ‘slow-dividing’ RGPs transition to quiescent aNSCs in the V-SVZ to allow persistence through the lifespan^6,7^. Using single-cell RNA-sequencing (scRNA-seq) of the developing neocortex, we characterized the transition to the RGP ‘slow-dividing’ state and predicted a number of genes which correlate with this transition^8^. One of these genes is *Lrig1*, which encodes the leucine-rich repeats and immunoglobulin-like domains protein 1 (LRIG1).

LRIG1 is a transmembrane protein that contains 15 leucine-rich repeats, 3 immunoglobulin-like domains and a short intracellular domain^9^. LRIG1 has been shown to interact with and negatively regulate the members of the ErbB family, including epidermal growth factor receptor (EGFR) by promoting its degradation via the action of c-Cbl^10,11^. Functionally, LRIG1 has also been proposed to act as a marker and regulator of a variety of quiescent stem cell populations including epidermal and intestinal stem cells acting through reduction of EGFR signalling^12,13^. Based on the correlation between *Lrig1* and the transition to the ‘slow-dividing’ state^8^ and its known role in quiescent stem cell populations we characterized the role of LRIG1 in RGPs in the developing neocortex and found that LRIG1 acts through EGFR to inhibit proliferation in RGPs to enable them to slow their proliferation and thus transition to a portion of aNSCs^14^. Emerging evidence suggests LRIG1 also plays a role in proliferation of aNSCs^15,16^, however EGFR is known to be expressed in actively dividing aNSCs and TAPs and is not found in high levels in quiescent aNSCs^17^. Because of the lack of expression of EGFR in quiescent aNSCs, how LRIG1 may control proliferation (or cell cycle re-entry from quiescence) of NSCs in the adult brain is not yet clear. Here we aimed to address this question and found that loss of LRIG1 leads to increased proliferation of aNSCs without significant impacts on EGFR signalling and neuronal progeny likely via the dysregulation of the transforming growth factor beta signalling (TGF*β*) superfamily of signalling pathways. Moreover, we show that LRIG1 directly binds receptors for both the TGF*β* and bone morphogenic protein (BMP) branches of these signalling pathways in addition to the TGF*β*1 ligand. Finally, we show that LRIG1 acts at the cell surface to facilitate SMAD phosphorylation. Collectively, these studies reveal LRIG1 as an important regulator of aNSC proliferation capable of acting broadly on TGF*β* superfamily signalling pathways.

## Methods

### Mice

*Lrig1* constitutive knockout mice were generated by crossing *Lrig1*Cre^ERT2^ (Jackson Strain No. 018418, *Lrig1*^tm1.1(cre/ERT2)Rjc/J^) heterozygous mice on the 129-Elite strain background (Charles River Canada, strain code 476) together to produce homozygous knockout mice as described in ref. ^14^. Genotypes were confirmed post-weaning from ear notch genomic DNA (isolated with DirectPCR Lysis Reagent (Ear, Cat # 401-E)) using PCR primers and Jackson Protocol 26202 exactly as described. For all experiments female and male mice were randomly assigned to experimental groups and analyzed at ages of 8-weeks, 15-weeks and 24-weeks. Specific timepoints are noted in the figure legends and captions. Mice used in this work all followed a 12 h light/dark cycle and had *ad libitum* access to water and rodent chow. Mice did not demonstrate any visible signs of being immunocompromised or display any obvious behavioural phenotypes. All animal work conducted followed policies formed by the Canadian Council of Animal Care and approved by the Local Animal Care Committee at the University of Toronto and The Hospital for Sick Children.

### Immunohistochemistry (IHC)

Frozen tissue sections were dried for 1 h at room temperature or 30 min at 37 °C, then rehydrated in phosphate buffered saline (PBS, 137 mM NaCl, 2.7 mM KCl, 10 mM Na_2_HPO_4_, 1.8 mM KH_2_PO_4_) for 10 min. Subsequently, sections were blocked and permeabilized in a solution of 5% BSA and 0.3% Triton X-100 in PBS. Following blocking, sections were incubated in primary antibody diluted in 1:1 PBS to blocking solution overnight, at 4 °C in a humidified chamber. The following day sections were washed in PBS three times for 5 min, then incubated in diluted secondary antibody for 2 h at room temperature. Afterwards, sections were washed three times in PBS for 5 min per wash. Finally, sections were counterstained for 5 min at room temperature in 0.5 *μ*g/mL Hoechst 33258 (Sigma-Aldrich), washed with PBS and then mounted with PermaFluor mountant (Thermo Scientific).

### EdU treatment and staining

5-ethynyl-2’-deoxyuridine (EdU, Toronto Research Chemicals Cat #: E932175) was dissolved in PBS and injected intraperitoneally three times two hours apart at a dose of 50 mg/kg to 15-week-old *Lrig1* WT and KO mice. After 3 weeks brains were collected and prepared for IHC as described below. EdU+ cells were detected using the Click-it EdU Alexa 488 kit (Invitrogen, Cat #: C10337) following the manufacturers instructions and then stained with Hoechst 33258. EdU+ cells were counted from five sections per brain.

### Proximity Ligation Assay (PLA)

Tissue sections from 15-week-old *Lrig1* WT and KO mice were prepared as described below, blocked and incubated overnight with 1:100 anti-LRIG1 and anti-BMPR-1B (1:100 see Supplementary Table S3) antibodies as described above for IHC. The next day sections were washed three times for 10 min with Wash Buffer A (0.01 M Tris, 0.15 M NaCl and 0.05% Tween-20) and then incubated with the Duolink In Situ PLA Probe Anti-Goat

PLUS (Sigma-Aldrich) and Duolink In Situ PLA Probe Anti-Mouse MINUS Affinity purified Donkey anti-mouse IgG (H + L) (Sigma-Alrich, See Supplementary Table S4) for 1 hour at 37 °C in a humidified chamber. Following washing of the PLA probes, Ligation and Amplification was carried out as described by the manufacturer using the Duolink In Situ Detection Reagents kit (Sigma-Aldrich) and nuclei stained for Hoechst 33258.

### Tissue collection for western blotting and bulk RNA sequencing

The periventricular area containing the V-SVZ from *N* = 2 female and *N* = 2 male *Lrig1* KO and *N* = 3 female and *N* = 1 male WT 8-week old brains was dissected as described in ref.^18^ from both hemispheres of each brain and stored at −80 °C until needed. One hemisphere sample was used to isolate RNA as described below. The other hemisphere sample was lysed and western blotted for LRIG1 and pEGFR as described previously^14^.

### Tissue preparation for IHC

*Lrig1* KO and WT mice were anesthetized with 2–3% inhaled isoflurane and then perfused transcardially with PBS followed by 4% paraformaldehyde (PFA). Brains were then dissected, post-fixed overnight in 4% PFA and then cryoprotected in 30% sucrose in PBS for 24 h. Tissues were then embedded with O.C.T (Fisher Healthcare™ Tissue-Plus™ O.C.T. Compound, Cat # 23-730-571) and sections were cut 18 *μ*m thick in the coronal plane using a Thermo Fisher Scientific HM525 NX cryostat at −20 °C. Sections were collected on glass slides (Fisherbrand Superfrost Plus Microscope Slides, Cat #1255015) coated with gelatin and stored frozen until use.

### Antibodies

All primary and secondary antibodies used for western blot are listed in Supplementary Tables S1 and S2, while those used for IHC/PLA are listed in Supplementary Tables S3 and S4.

### Plasmids

For all experiments, plasmid constructs were used following endotoxin-free maxipreps using a Qiagen EndoFree® Plasmid Maxi Kit or a ZymoPURE™ II Plasmid Maxiprep Kit. Plasmid DNA concentration was determined using a NanoDrop 2000 (ThermoFisher). All plasmids used in this study are listed in Supplementary Table S5.

### Imaging and microscopy

Images were collected using a Zeiss Spinning Disk confocal microscope system or a Zeiss AxioImager M2 microscope system with a Calibri LED light source. Images were acquired using Z-stacks (with the apotome engaged in the case of the AxioImager) and staked tiles imaging set-up using Zen Blue software. All images were acquired with Z-stack sizes ranging between 15-25 slices depending on the dataset analyzed. Images shown were produced using the orthogonal projection feature implemented in Zen Blue.

### Quantification of IHC

For all cell count analysis on acquired images from V-SVZ, counting was done using ImageJ. For cell counts in the V-SVZ, only visibly immunostained (positive) cells along the dorsal and ventral portions of the lateral wall (LW), closest to the ventricle (periventricular area) were counted. To differentiate between the dorsal and ventral areas of the LW, these regions were measured using the line drawing and measurement tools in ImageJ along the length of the entire LW. To assess proliferation, neuronal progeny and pEGFR-positive cells in the V-SVZ, the top 1/3^rd^ of the length of the LW was considered the dorsal portion. The bottom 2/3 measurement of the LW was considered the ventral portion. To obtain a cell count value for each individual brain, positive cells counted from three anatomically matched 18 μm thin coronal sections containing the V-SVZ LW region were totaled and averaged. For the proportion of proliferating (Ki67 + ) cells, a percent cell count was used to represent the data by dividing the number of SOX2 + GFAP+Ki67 + /SOX2 + GFAP+ cells. For the number of DCX-positive and pEGFR-positive cells the total number of cells counted per ventricle area was used to represent these data. For cell counts in the OB, positive cells in the GCL, GL and MCL were counted using three anatomically 18 μm thick coronal sections of the whole OB from each mouse brain. To create a cell count value for each individual brain, counts from all three sections were totaled and averaged. For the number of CalB or CalR-positive interneurons, the total number of cells in each layer were counted and used to represent these data. pSmad2 and pSmad1/5/9 positive cells only in the V-SVZ were counted on the entire ventral portion of the LW of the LV. Three coronal sections from each brain which were anatomically matched in order of contain the LW area of the V-SVZ and were used for cells count analysis, where the total positive cells from each coronal section were then averaged.

### RNA extraction and bulk RNA sequencing

Following RNA isolation using a RNeasy Plus Mini Kit (Qiagen, Cat#:74134) from tissue isolated as described above, polyA selected mRNA next generation sequencing libraries were prepared using the NEBNext® Ultra™ II DNA Library Prep Kit and sequenced on one lane of an Illumina Novaseq SP flow cell achieving ~40–50 million reads per sample. Following sequencing, FASTQ files were generated with bcl2fastq2 v2.20. Library preparation, sequencing and FASTQ file production were done by The Centre for Applied Genomics (TCAG) at the Hospital for Sick Children. FASTQ files were then used as input for Salmon^19^ for alignment to the mouse genome (Gencode m29) and for read quantification. Deseq2 as implemented in R and was used for normalization and differential gene expression analysis^20^.

### Immunoprecipitation assays

Immunoprecipitation (IP) assays were done similar to those described in ref. ^21^. Neuro-2a cells (N2a) (ATCC Cat No: CCL-131) were used for all IP experiments which were cultured in Dulbecco’s modified Eagles medium (DMEM) containing high levels of glucose supplemented with 1% penicillin-streptomycin and 10% fetal bovine serum (FBS). Transfections of N2a cells were carried out using Poly-Jet reagent (SignaGen Laboratories; Cat# SL100688) 24 h after plating 400,000 cells per well of 6 well plate. Flag-tagged pCMV expression vectors containing either the TGF*β*R1, TGF*β*RII and BMPRI were used to co-transfect N2a cells with *Lrig1* pCMV-overexpression plasmid using PolyJet In Vitro DNA Transfection Reagent (SignaGen Laboratories; Cat# SL100688). A mock condition with no plasmids was used as a negative control and BMPRI was used as a positive control^21^. N2a cells were lysed after 48 h in TNTE buffer containing 0.5% Triton-X-100 (150 mM NaCl, 50 mM Tris pH 7.4, and 1 mM EDTA)^21^. Anti-Flag Magnetic beads (*Selleckchem* Cat#: B26101) were used to carry out all immunoprecipitations. The beads and sample mixtures were incubated on a tube rotator at 4 °C for 2 h. Supernatant was then discarded and the beads were washed three times with wash buffer (TNTE buffer containing 0.1% Triton-X100) on a magnetic separation rack. Following the final wash, magnetic beads were eluted using 1x SDS-PAGE loading buffer heated at 70 °C for 10 min. 10% of the samples prior to immunoprecipitation were collected for a load condition in 5x SDS Sample Loading Buffer (10% SDS, 500 mM DTT, 50% Glycerol, 250 nM Tris-HCl, 0.5% bromophenol blue dye, pH 6.8). Western blotting was performed as described previously^14^ using anti-flag antibody (DYKDDDDK Tag Antibody Cell Signalling Technology Cat#: 2368) to detect the TGF*β*R1, TGF*β*RII and BMPRI receptors and LRIG1 antibody (R&D Systems, Cat#:3688) to detect LRIG1. GAPDH antibody was used as an internal reference to normalize protein expression levels.

### Far western assay

LRIG1 ectodomain (ECD, Cat#: 3688-LR-050 from R&D Systems) concentrations of 2.5 *μ*g/mL, 0.25 *μ*g/mL, and 0.025 *μ*g/mL were immobilized on a nitrocellulose membrane in duplicate. 2.5% Bovine serum albumin and 1xPBS were used as negative controls. Once dried, the blot was incubated at room temperature (RT) in blocking buffer (2% BSA in PBS-T (1xPBS containing 0.1% Tween)) for 30 min. 1.2 ug of biotinylated human TGF*β*1 (Avi- Tag, Biotin-Labeled, BPS Bioscence Cat #. 100843) was then added to blocking buffer on the blot and incubated for one hour at RT. A 1:5000 dilution of Streptavidin-HRP in blocking buffer was added for 30 min followed by 3 × 5 min and 3 × 15-minute washes with PBS-T. The blot was then developed with Bio-Rad Clarity Western enhanced chemiluminescence (ECL) Detection Reagent on a Syngene G:Box chemiluminescence imager.

### Bio-layer interferometry

The binding affinity of LRIG1 extracellular domain (ECD) to TGF*β*1 was measured using a BLItz instrument. LRIG1 ECD in 1xPBS was used as the analyte and biotinylated-TGF*β*1 was used as the bait. Streptavidin-coated sensors were hydrated in BLI rehydration buffer (PBS, 0.5 mg/mL BSA, and 0.02% (v/v) Tween-20). The biotinylated bait protein was diluted in kinetics buffer (PBS, 0.5 mg/mL BSA, 0.02% (v/v) Tween-20) to a final concentration of 12.4 *μ*g/mL. The bait was immobilized on a streptavidin-coated biosensor tip for 30 s. Next, the analyte concentrations were diluted in kinetic buffer in order to obtain concentration of LRIG1 ECD of 1 μM, 750 nM, 500 nM, 100 nM. The binding association was measured over a period of 120 s with subsequent dissociation measured after immersion of the biosensor tip into kinetic buffer for another 120 s. The data were analyzed and sensorgrams were step corrected, reference corrected and globally fit to a 1:1 binding model. Dissociation constants (K_D_) were calculated using BLItz Pro Version 1.1.0.16 and the average of two independent determinations is reported here.

### *Lrig1* KO and Ctrl clonal Neuro-2a cell lines

N2a cells were transfected with guide RNA’s #1, #2, or LacZ control contained in the pU6-(Bbsl)_CBh_Cas9_T2A_mCherry plasmid (as described previously^14^ in using Lipofectamine STEM, according to manufacturer’s protocol in 6 well plates. Two days following transfection, cells were replated into 96 well plates at a concentration of 0.5 cells/200 *μ*l media in each well. Cells were allowed to grow for 3 weeks and wells containing individual colonies that expressed red fluorescence from mCherry were selected and propagated further to produce clonal cell lines. Three control lines and four LRIG1 KO lines were selected following western blotting for LRIG1 to confirm loss of the LRIG1 protein and used for the experiments described here.

In the case of TGF*β*1 and BMP4 treated non-transfected Ctrl N2a clonal cell lines and *Lrig1* KO N2a clonal cell line experiments, cells were plated at 90,000 cells/well in DMEM media (described above) in 24-well plates. Two days later the media was changed to DMEM containing 0.1% FBS for serum starvation overnight. The following day, 10 ng/ml TGF*β*1 (Cedarlane; Cat# 781802) or BMP4 (Peprotech Cat#315-27) ligands suspended in DMEM containing 0.1% FBS. The later of which on its own was used as the control. Following 30 min at 37 °C, the cells were collected using 50 *μ*L of lysis buffer containing, tris-buffered Saline, pH 7.4, 0.5% b-octyl-D-glucopyranoside, 0.5% Triton X-100, 1 mM NaF, 1 mM ß-glycerophosphate, 1 mM Na_3_VO_4_ and 1 cOmplete, mini, EDTA-free protease inhibitor tablet per 10 ml. Protein assay was done with the samples using the DC Assay (Bio-Rad) and then equal amounts of protein were used for western blots to assess the pSmad2/3, total Smad2, pSMAD1/5/9 and total SMAD1 along with GAPDH (as a loading control) and LRIG1 (to confirm genotypes).

Since N2a cells express low levels of TGF*β*RII, we used TGF*β*RI and TGF*β*RII constructs to express these receptors in Ctrl and *Lrig1* KO clonal N2a cells followed by treatment with TGF*β*1 to assess the response of this signalling pathway in the context of loss of LRIG1. To do so, 1 *μ*g of DNA per well, using PolyJet of TGF*β*RI and TGF*β*RII were transfected one day after plating 90,000 cells per well of each of the Ctrl and *Lrig1* KO clonal N2a cell lines. 24 h after transfection, the cells were transitioned into DMEM containing 0.1% FBS media. The following the *Lrig1* KO and WT clonal cell lines were treated with 10 ng/ml per well of TGF*β*1 for 30 min at 37 °C, lysed, and protein concentrations determined as above. Western blotting for pSmad2/3, total Smad2 and GAPDH were then performed.

### Neurosphere cultures

Neurospheres were grown essentially as described in ref. ^18^ using NeuroCult Mouse&Rat Basal Medium containing NeuroCult Proliferation Supplement (STEMCELL Technologies), FGF2 (Corning), EGF (STEMCELL Technologies), 0.2% heparin (Sigma-Aldrich, Cat #: H4784) and 1% penicillin-streptomycin from brains of 15 week old *Lrig1* WT and KO mixed male and female mice (*N* = 2 male and *N* = 1 female KO mice, *N* = 2 male and *N* = 2 female WT mice). Following seven days of growth of secondary spheres, spheres from each brain were gently distributed into two wells of a 24-well plate and treated with BMP4 as described above without changing the culture media. Cells were then collected by centrifugation and western blots were performed as described above.

### Analysis of scRNA-sequencing data

Data previously published by Mizrak et al.^22^, from lateral wall cells of 8–10 week old mice was obtained from NCBI GEO accession number: GSE109447. Specifically, the 13,055 cell data set was reduced to just the lateral wall cells and then was processed using Seurat (version 4.4.0) as implemented in R (version 4.2.1). Cells with less than 200 genes expressed in at least three cells were removed. The data were then normalized and scaled, principal components were computed and clustering was performed with 25 principal components and a resolution of 0.8. UMAP plots were created using the DimPlot and FeaturePlot functions as implemented in Seurat.

### Statistics and reproducibility

**Fig. 1:**
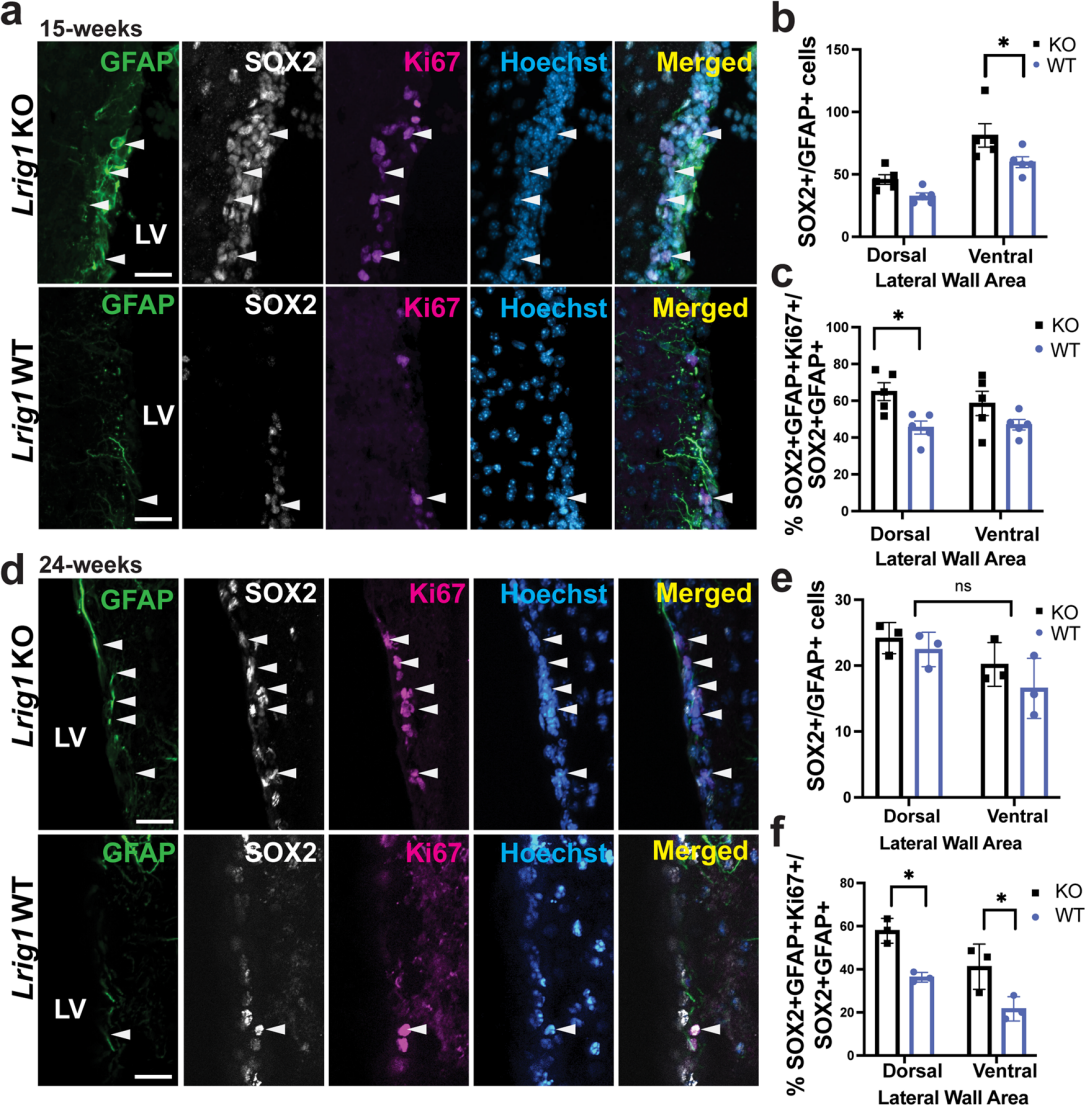
*Lrig1* KO results in increased proliferation of NSCs in the V-SVZ. **a** Immunostaining of the lateral wall of 15-week old *Lrig1* KO and WT mice for GFAP, SOX2, Ki67 and merged with Hoechst to label nuclei. Sox2/GFAP-double positive cells allows identification of NSCs. Ki67/SOX2/GFAP-triple positive cells represent proliferating NSCs. Arrowhead indicate triple positive cells. Quantification of the number of SOX2/GFAP-double positive NSCs (**b**) and proportion of proliferating Ki67-positive NSCs (**c**) along the dorsal and ventral portions of the lateral wall in 15-week old *Lrig1* KO and WT mice. **d** Immunostaining of the lateral wall of 24-week old Lrig1 KO and WT mice for GFAP, SOX2, Ki67 and Merged with Hoechst. Quantification of the number of SOX2/GFAP-double positive NSCs (**e**) and proportion of proliferating Ki67-positive NSCs (**f**) along the dorsal and ventral portions of the lateral wall in 24-week old *Lrig1* KO and WT mice. Scale bars represents 25 µm. Error bars indicate S.E.M. For (**a**–**c**, *N* = 5 per group, and for **d**–**f**, N = 3 per group. Source data for graphs are included in Supplementary Data 2.

**Fig. 2:**
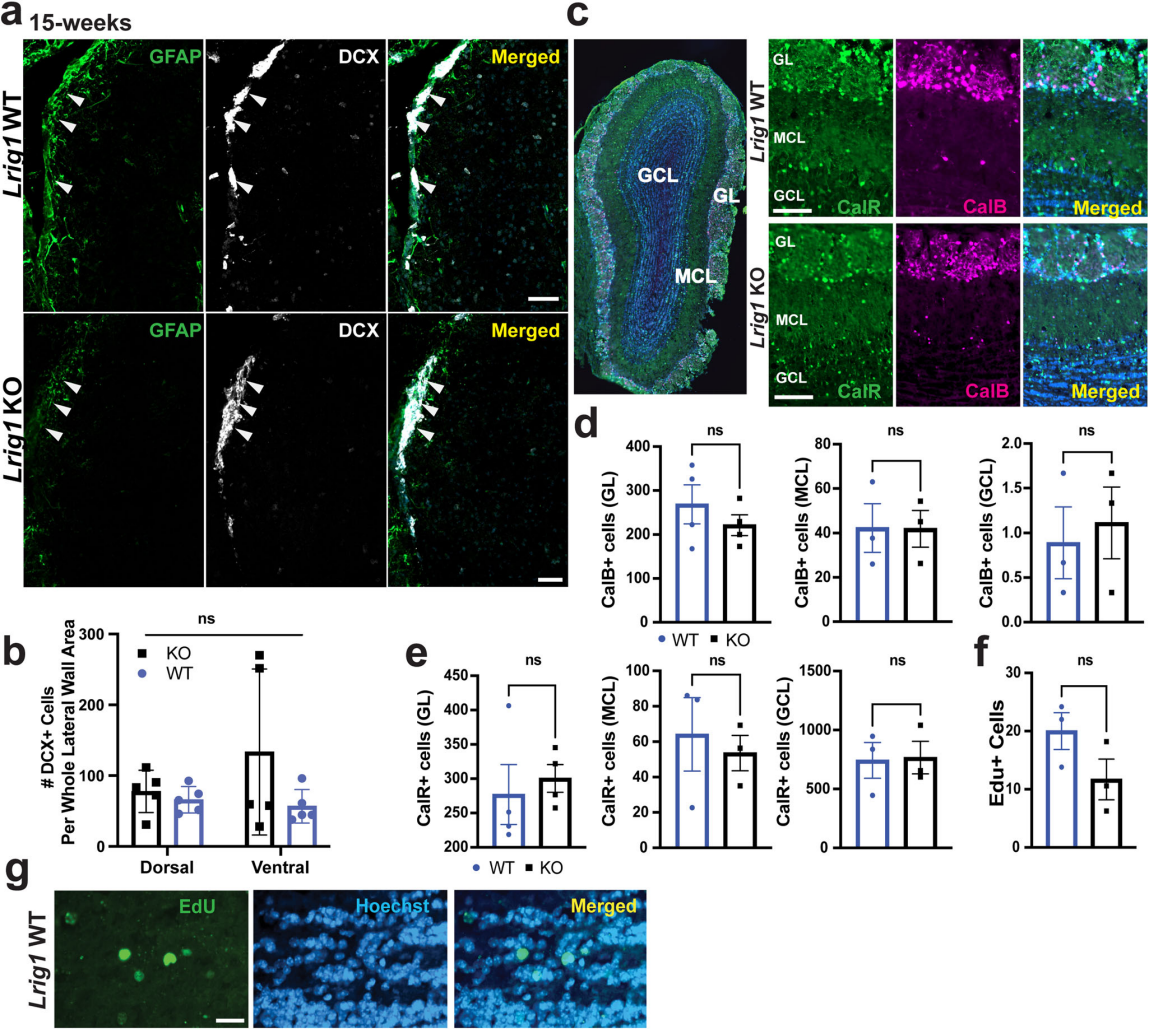
Enhanced proliferation in the context of *Lrig1* KO does not impact neuronal progeny. **a** Immunostaining of coronal sections through the lateral ventricle of 15-week old *Lrig1* KO and WT mice for DCX to mark new-born neurons/neuroblasts and GFAP to label ventricular cells merged with Hoechst. **b** Quantification of the number of DCX-positive cells in the dorsal and ventral portions of the lateral wall. **c** Representative immunostaining of Olfactory Bulb (OB) sections with antibodies for Calretinin (CalR) and Calbindin (CalB) and merged with Hoechst at low magnification (left, indicating the Granule Cell Layer (GCL), the Mitral Cell Layer (MCL) and the Glomerular Layer (GL)) and at high magnification (right) from *Lrig1* and KO mice. **d** Quantification of the number of CalB-positive cells in each of the granule cell (GCL), mitral cell (MCL) or glomerular layers (GL), (**e**) Quantification of the number of CalB-positive cells in the GCL, MCL and GL of the OB. **f**, **g** Quantification of the number of EdU-positive cells in the olfactory bulb (**f**) and representative staining of EdU-positive cells with Hoechst from an *Lrig1* WT brain. Scale bars represent 50 µm for (**a**), 100 µm for (**c**) and 20 µm for (**g**). Error bars represent S.E.M. For (**a**, **b**, *N* = 5 per group and for **c**–**f**, *N* = 3 per group. Source data for graphs are included in Supplementary Data 2.

**Fig. 3:**
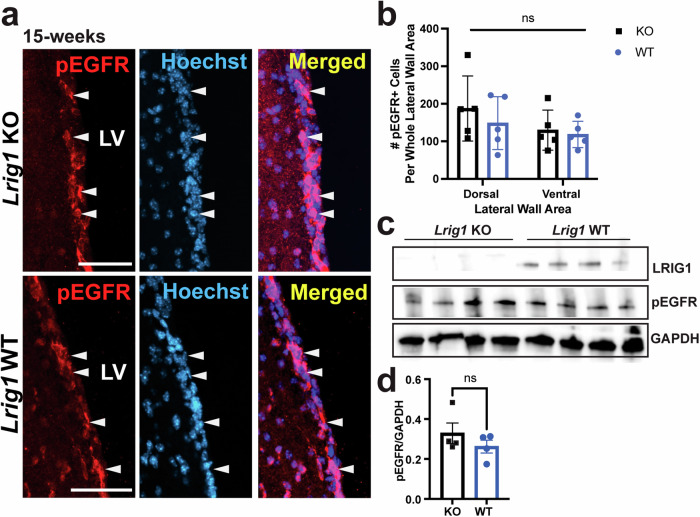
pEGFR levels are unchanged in the V-SVZ in *Lrig1* KO and WT mice. **a** Immunostaining of coronal sections along the lateral wall of *Lrig1* KO and WT mice with antibodies for phosphorylated EGFR (pEGFR) and merged with Hoechst. **b** Quantification of the number of pEGFR-positive cells in the dorsal and ventral portions of the lateral wall of the V-SVZ. **c** Western blots using antibodies for LRIG1, pEGFR and GAPDH (loading control) from periventricular tissue dissected from one hemisphere per animal from 8-week old *Lrig1* KO and WT mice See also Supplementary Fig. S2d. **d** Quantification of the western blot data from (**c**) for pEGFR relative to GAPDH. Scale bar represents 50um. Error bars represent S.E.M. For (**a**, **b**, *N* = 5 per group and for **c**, *N* = 4 per group. Source data for graphs are included in Supplementary Data 2.

GraphPad Prism Version 9.3.1 software was used to perform all statistical analyses and create graphical representations. For statistical analyses in Figs. 1–3, a two-way ANOVA and Tukey’s Honestly Significant Difference (HSD) *post-hoc* test were used to compare data between the different experimental groups present in each paradigm (ex. KO vs. WT), and the area of the LW being analyzed (ex. dorsal vs ventral). In Fig. 5, a two-tailed Student’s t-test in order to compare the differences between *Lrig1* KO and WT groups. In Fig. 7 and Supplementary Fig. S3, the results from the four conditions were averaged from two independent experiments and normalized to total SMAD2/3 for the TGF*β*R transfections or total SMAD1 for the BMP experiments. Subsequently, a one-way ANOVA followed by a Šídák post-hoc test was used to compare groups. In all figures, values were reported as means. To create error bars, means were represented as the standard error of the mean (S.E.M). Experimental data was denoted as statistically significant at P values less than 0.05 (P < 0.05). In all the figures, *p < 0.05, **p < 0.01, ***p < 0.001 and ns = not significant.

## Results

### Loss of LRIG1 enhances NSC proliferation without impacting neuronal progeny

To begin to assess the role and mechanism of LRIG1 in adult neural stem cells we produced *Lrig1* knockout (KO) mice by crossing mice heterozygous for Cre-recombinase (CreERT2) knocked into exon 1 of the endogenous *Lrig1* locus. In so doing, mice homozygous for CreERT2 serve as a constitutive knockout (KO) as previously described in ref. ^23^ and homozygous wild-type allele littermates act as controls. Once mice reached the age or 15-weeks (2-3 months), brains were collected, sectioned coronally through the LV and stained for SOX2 and GFAP, which when co-expressed allow the identification of aNSCs in the V-SVZ. As the dorsal and lateral areas of the V-SVZ are well known to have different developmental origins and produce different types of OB interneurons^7,24^ we analyzed the number of NSCs in these areas separately. We found significantly increased number of NSCs in the ventral area of the lateral wall of the V-SVZ and a trend towards increased NSCs in the dorsal area in *Lrig1* KO mice (Fig. 1a, b). Further we considered whether NSCs in these areas were proliferating more robustly and found that the proportion of Ki67-positive NSCs, marked by GFAP/SOX2, was significantly increased in the dorsal area of the V-SVZ (Fig. 1a, c). To confirm the robustness of these NSC and proliferation phenotypes and ask how LRIG1 loss may impact NSCs with advancing age we analyzed a second cohort of mice at 6 months of age (24-weeks). Here, we found no significant change in the number of NSCs in both the dorsal and ventral areas of the V-SVZ, although the trends were similar to the 15-week cohort (Fig. 1d, e). As was the case at 15 weeks, we identified a robust increase in proliferating Ki67-positive NSCs in both the dorsal and ventral areas at 6 months of age (Fig. 1f). Collectively, these data suggest that NSCs and proliferating NSCs are enhanced in *Lrig1* KO mice.

Having, identified that proliferation of NSCs is enhanced in *Lrig1* KO mice, we then asked whether neuronal progeny are also impacted. To do this, we used three different approaches. First, we stained V-SVZ sections from 15-week old *Lrig1* KO and WT mice for the new-born neuron and neuroblast maker doublecortin (DCX) and found no difference in the number of DCX-positive cells in either the dorsal or ventral areas (Fig. 2a, b). Secondly, we stained olfactory bulb sections from these mice for markers of olfactory bulb (OB) interneurons Calbindin (CalB) and Calretinin (CalR) which are derived from NSCs in the ventral and dorsal areas of the V-SVZ, respectively^7,25^ (Fig. 2c). We found no significant difference in the numbers of CalB (Fig. 2d) or CalR-positive (Fig. 2e) OB interneurons in the *Lrig1* KO mice in any of the granule cell (GCL), mitral cell (MCL) or glomerular layers (GL). Finally, to eliminate any potential developmental confound on OB interneurons and specifically measure OB interneurons born at this time, we treated 15-week-old *Lrig1* KO and WT mice with Ethynyl-2’-deoxyuridine (EdU) and then collected brains 3-weeks later. No significant difference in the number of EdU positive cells in the OB were found (Fig. 2f, g), although there was a trend towards reduced EdU positive cells. Such a potentially lower number of EdU positive cells could result from dilution of the EdU in *Lrig1* KO NSCs which have increased proliferation. Thus, we find that even though proliferation of adult NSCs in the V-SVZ is enhanced in *Lrig1* KO mice this does not lead to increased numbers of neuronal progeny early on as they are produced in the V-SVZ or later as they arrive at the OB.

### Increased NSC proliferation does not occur through dysregulated EGFR signalling

Next, we turned our attention to the mechanism underlying the enhanced proliferation of NSCs in the context of loss of LRIG1. In our previous work, we showed that in the developing brain LRIG1 likely functions through EGFR to dampen proliferation. However, in published adult V-SVZ single-cell RNA-sequencing (scRNA-seq) data, *Egfr* is more highly expressed in activated NSCs and transit-amplifying progenitors (TAPs) as opposed to the more quiescent NSCs where *Lrig1* is expressed (Supplementary Fig. S1a, b). Moreover, EGFR is a common marker used to identify and isolate activated NSCs and TAPs. Given these considerations, the potential for LRIG1 to function through EGFR is less likely. To specifically address whether LRIG1 functions through EGFR in adult NSCs we stained coronal sections from 15-week-old *Lrig1* KO and WT mice and analyzed the number of phosphorylated EGFR-positive (and thus activated; pEGFR) cells along the ventricle (Fig. 3a) and found no significant difference in either of the dorsal or ventral areas of the lateral wall (Fig. 3b). To further confirm these findings, we microdissected the periventricular area, encompassing the V-SVZ, from one hemisphere of 8-week old *Lrig1* KO and WT mice and then performed western blots for LRIG1, pEGFR and GAPDH as a loading control. We observed a very clear loss of LRIG1, thereby confirming the genotypes of these mice and again, identified no differences in the levels of pEGFR (Fig. 3c, d & Supplementary Fig. S2d). Finally, we also assessed ETS Like-1 protein (ELK1), which acts downstream of activated EGFR and found no difference in activated pELK1 in 15-week-old *Lrig1* KO mice compared to WT (Supplementary Fig. S1e). These data strongly suggest that LRIG1 functions through an EGFR-independent pathway in adult NSCs.

### RNA sequencing identifies downregulation of TGF*β* superfamily signalling pathway downstream targets

**Fig. 4:**
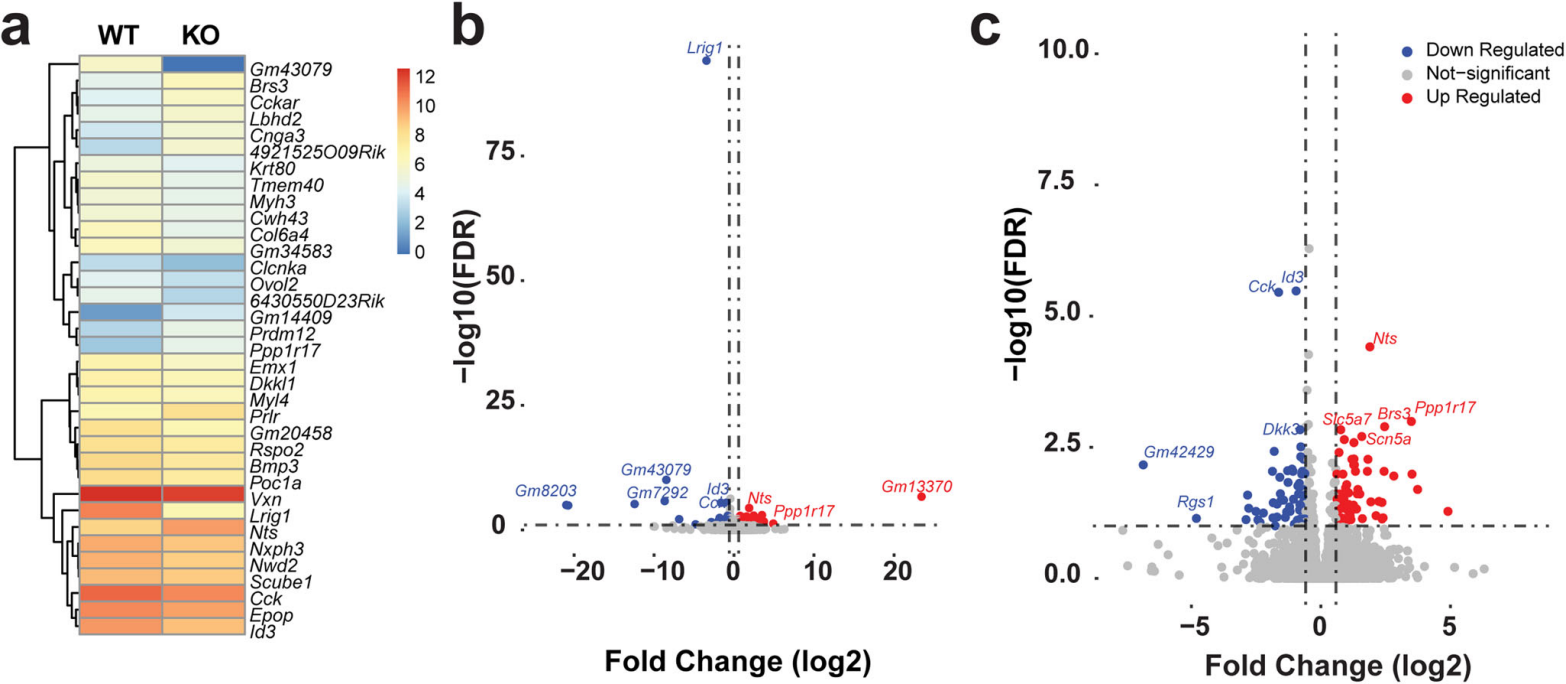
Bulk RNA-Sequencing from the periventricular area of 8-week old *Lrig1* KO and WT mice. mRNA was isolated and sequenced from one hemisphere of *N* = 4 *Lrig1* KO and WT mice at 8-weeks of age. **a** Heatmap of average expression of top 35 genes (25 down-regulated and 10-upregulated.) from the WT and KO groups. **b**, **c** Volcano plot of all 217 differentially expressed genes (adjusted *p*-value > 0.1) where the vertical line indicates ±1.5-fold change and blue dots are down-regulated and red dots up-regulated at this significance and fold change level. Grey dots have fold changes lower than ±1.5-fold change. The scale in (**c**) is limited to ±8 log2-fold change and up to a -log10(FDR) *p*-value of 10. FDR indicates false-discovery rate.

In an attempt to identify what EGFR-independent pathway LRIG1 could be acting through to control proliferation of adult NSCs, we collected bulk RNA-sequencing (RNA-seq) data from the microdissected periventricular tissue from the contralateral hemispheres of the 8-week old KO and WT mice used for western blotting above (Fig. 3c). Here we identified relatively subtle transcriptional changes with a total of 217 differentially expressed genes between these groups (adjusted *p*-value < 0.1, Fig. 4 and Supplementary Data 1). Importantly, the most significantly down-regulated gene was *Lrig1* (adjusted *p*-value of 7.6 × 10^−95^, Fig. 4b). Within this set of 217 differentially expressed genes we did not identify any obvious genes involved in EGFR mediated signalling but did identify genes such as *Id3*, *Id2* and *Bmp3* that are known upstream and downstream mediators of TGF*β* superfamily signalling pathways which include TGF*β* and BMP signalling. As shown in Fig. 4a, c, *Id3* was one of the most significantly down-regulated genes in these data (adjusted *p*-value of 3.3 × 10^−6^). As prior studies have demonstrated that LRIG1 can directly bind the BMP receptor^21^, our RNA-seq data shows the absence of EGFR pathway dysregulation and the down-regulation of some TGF*β*signalling pathway target genes, this would suggest that the TGF*β* superfamily signalling may be impaired in *Lrig1* KO mice. Moreover, ligands such as *Tgfb1* and *Bmp4* and receptors *Tgfbr1*, *Tgfbr2*, *Bmpr1a*, *Bmpr1b*, *Bmpr2* are all expressed in scRNA-seq profiling of similarly aged V-SVZ cell types (Supplementary Fig. S1c, d).

### pSMAD signalling is impaired in *Lrig1* KO mice

**Fig. 5:**
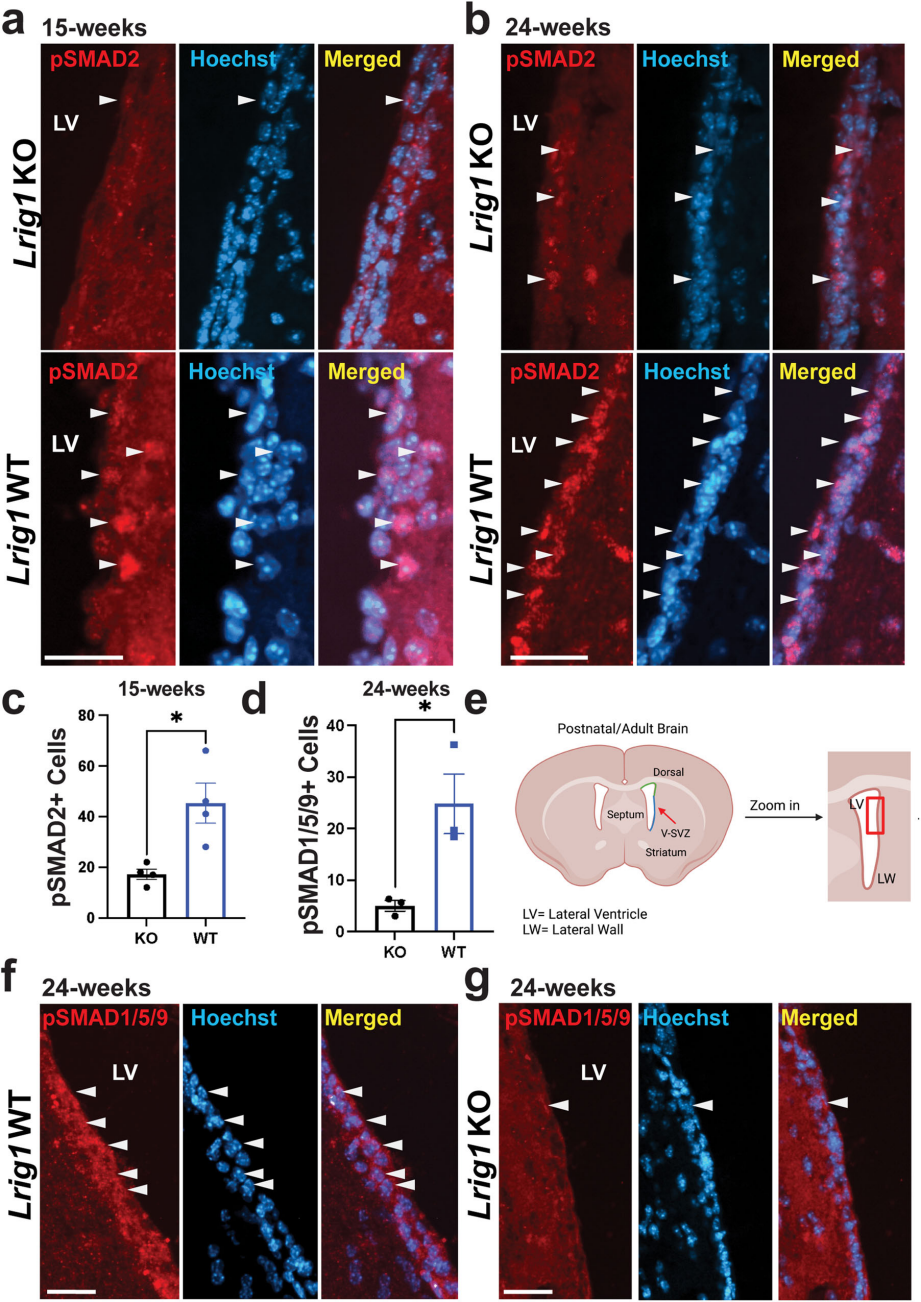
*Lrig1* KO results in decreased numbers of pSMAD-positive cells along the lateral wall of the V-SVZ. **a**, **b** Immunostaining of sections from the lateral ventricular wall (LW) with an antibody for phosphorylated SMAD2 (pSMAD2) from 15- and 24- week old *Lrig1* KO and WT mice merged with Hoechst. **c** Quantification of the number of pSMAD2-positive cells along the (LW) from (**a**). **d** Quantification of the number of phosphorylated SMAD1/5/9 (pSMAD1/5/9) positive cells along the LW in 24-week old *Lrig1* KO and WT mice. **e** Location of the area imaged in representative images of immunostaining for pSMAD1/5/9 and Hoechst. Created with BioRender.com (**f**, **g**). Scale bars represent 25 um. Error bars indicate S.E.M. For (**c**), *N* = 4 per group and for (**d**), *N* = 3 per group. Source data for graphs are included in Supplementary Data 2.

TGF*β* superfamily signalling can be divided into three separate branches: the TGF*β* activated branch, the BMP activated brand and the non-canonical pathway^26^. In both the TGF*β* and BMP branches of this superfamily, ligand binding to the TGF*β*-receptor or BMP receptors, respectively, leads to the phosphorylation of receptor SMADs, SMAD2/3 (TGF*β*) or SMAD1/5/9 (BMP) in mice. Once these receptor SMADs are phosphorylated they can bind SMAD4, translocate to the nucleus and induce gene expression. As the role of LRIG1 in these pathways is poorly understood and our data suggests that they may be dysregulated in adult NSCs, we considered both the TGF*β* and BMP branches of this superfamily. To do this, we first stained coronal section from *Lrig1* KO and WT sections from 15- (Fig. 5a, e) and 24-week old (Fig. 5b, e) mice for phosphorylated SMAD2 (pSMAD2) to assess TGF*β* signalling. Here, we found significantly fewer pSMAD2-positive cells along the lateral well at 15-weeks (Fig. 5c) and a similar trend at 24 weeks (Supplementary Fig. S4a) but this did not reach significance. To assess the impacts of LRIG1 on the BMP branch of this superfamily, we performed similar immunostaining using an antibody for phosphorylated SMAD1/5/9 (pSMAD1/5/9, Fig. 5e–g). Akin to the pSMAD2 data, we observed a significant decrease in the number of pSMAD1/5/9-positive cells along the lateral wall in both 15-week-old (Supplementary Fig. S4b) and 24-week old *Lrig1* KO mice (Fig. 5d). On balance, these pSMAD data suggest that LRIG1 may be acting broadly to facilitate signalling in the TGF*β* superfamily.

### LRIG1 directly interacts with both BMP and TGF*β* receptors and TGF*β*1 ligand

**Fig. 6:**
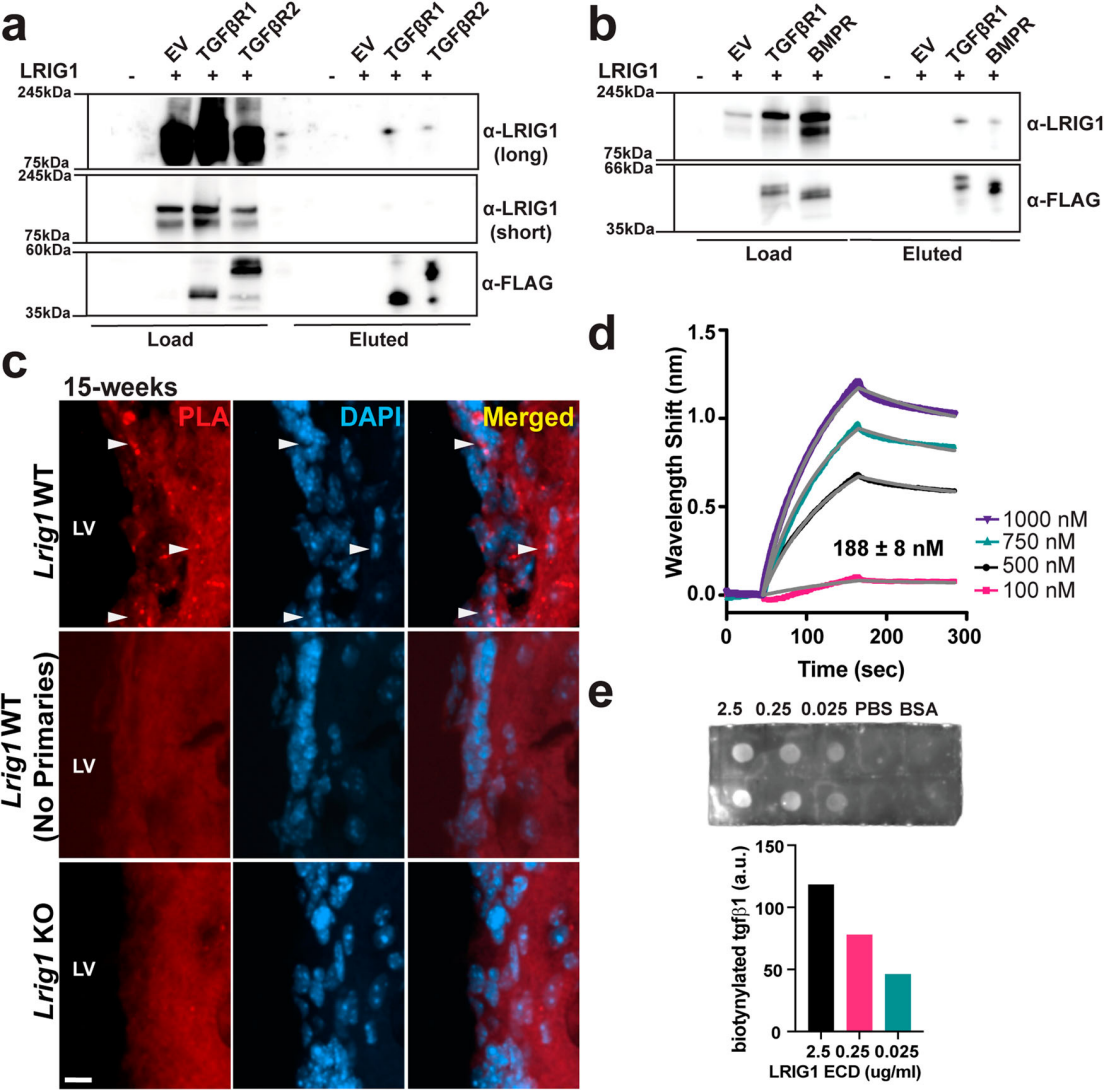
LRIG1 interacts directly with TGF*β*RI, TGF*β*RII, BMPRI and TGF*β*1 in vitro*.* **a** Immunoprecipitation of FLAG-tagged TGF*β*R1 and TGF*β*R2 and probed using antibodies for LRIG1 (long and short exposures) and FLAG by western blot. Load indicates a sample removed following cell lysis and prior to loading on the anti-FLAG magnetic beads. Eluted indicates sample retained on the anti-FLAG magnetic beads. Data are representative of *N* = 3 independent experiments. See also Supplementary Fig. S2a, b (**b**) Immunoprecipitation of FLAG-tagged TGF*β*R1 and BMPR1B and probed using antibodies for LRIG1 and FLAG by western blot. Data are representative of *N* = 3 independent experiments. See also Supplementary Fig. S2c (**c**) Proximity Ligation Assay (PLA) using antibodies to LRIG1 and BMPR1B on *Lrig1* WT (with and without primary antibodies (Red) in addition to *Lrig1* KO mice along the lateral wall of the lateral ventricle (LV), scale bar indicates 10 µm. **d** Biolayer interferometry using biotinylated TGF*β*1 as the bait and the LRIG1 extracellular domain (ECD) in increasing concentrations as the analyte. Fitted curves used to measure K_D_ are shown as grey lin**e**s. **e** Far-western assay using decreasing amounts of LRIG1 ECD bound to the nitrocellulose membrane and detected by incubating with biotinylated TGF*β*1 and streptavidin HRP and quantification (right). For (**c**, **d**), data are representative of *N* = 2 independent experiments. Source data for graphs are included in Supplementary Data 2.

While LRIG1 has been shown to bind to the BMP receptors^21^, it’s ability to bind the receptors of the TGF*β* family more broadly has not been studied. Thus, we next carried out a series of immunoprecipitation assays using overexpression of mouse LRIG1 and FLAG-tagged TGF*β*R1 and TGF*β*R2 in mouse Neuro-2a (N2a) cells. When TGF*β*R1 and TGF*β*R2 were pulled down with anti-FLAG magnetic beads, we found that LRIG1 was pulled down by both of these receptor subunits suggesting that LRIG1 interacts directly with the TGF*β* receptor and perhaps more robustly with TGF*β*R1 than TGF*β*R2 (Fig. 6a & Supplementary Fig. S2a, b). We further extended these studies to the BMP receptor and performed similar experiments again using the FLAG-tagged TGF*β*R1 and an additional FLAG-tagged BMPR1B construct (Fig. 6b & Supplementary Fig. S2c). When TGFBR1 and BMPR1B were immunoprecipitated we identified that LRIG1 was also pulled down in similar amounts, confirming prior studies and suggesting that LRIG1 directly interacts with both TGF*β*- and BMP receptors with similar affinities (Fig. 6b). To assess whether the LRIG1 and BMPR1B interaction may also occur in vivo, we performed proximity ligation assays (PLA) on both *Lrig1* WT and KO mice at 15-weeks of age. We identify puncta that were only produced along the ventricle in the *Lrig1* WT and not KO animals or in WT animals not treated with primary antibodies (Fig. 6c), thus confirming the specificity of this approach and the close proximity of LRIG1 and BMPR1B in vivo.

Binding directly to the multiple TGF*β* superfamily receptors would put LRIG1 in proximity to the ligand when these pathways are activated, thus we tested whether LRIG1 directly binds to a biotinylated form of TGF*β*1 using two orthogonal approaches. First, we used biolayer interferometry (BLI) wherein the biotinylated TGF*β*1 acted as the bait on the probe with increasing amounts of the LRIG1 extracellular domain (ECD) from 100 to 1000 nM acting as the analyte. In so doing, we found that LRIG1 is capable of binding directly to TGF*β*1 with a dissociation constant (K_D_) of 188 ± 8 nM (Fig. 6d). In the second approach, we spotted the LRIG1 ECD on nitrocellulose membrane in decreasing amounts and then probed the membrane in a far-western approach with the biotinylated Tgfß1 and found as the case in the BLI data that LRIG1 is able to directly bind to TGF*β*1 (Fig. 6e) and thus collectively LRIG1 binds to multiple TGF*β* superfamily pathway receptors and to the TGF*β*1 ligand itself.

### LRIG1 acts early on to activate TGF*β* pathways by facilitating SMAD phosphorylation

**Fig. 7:**
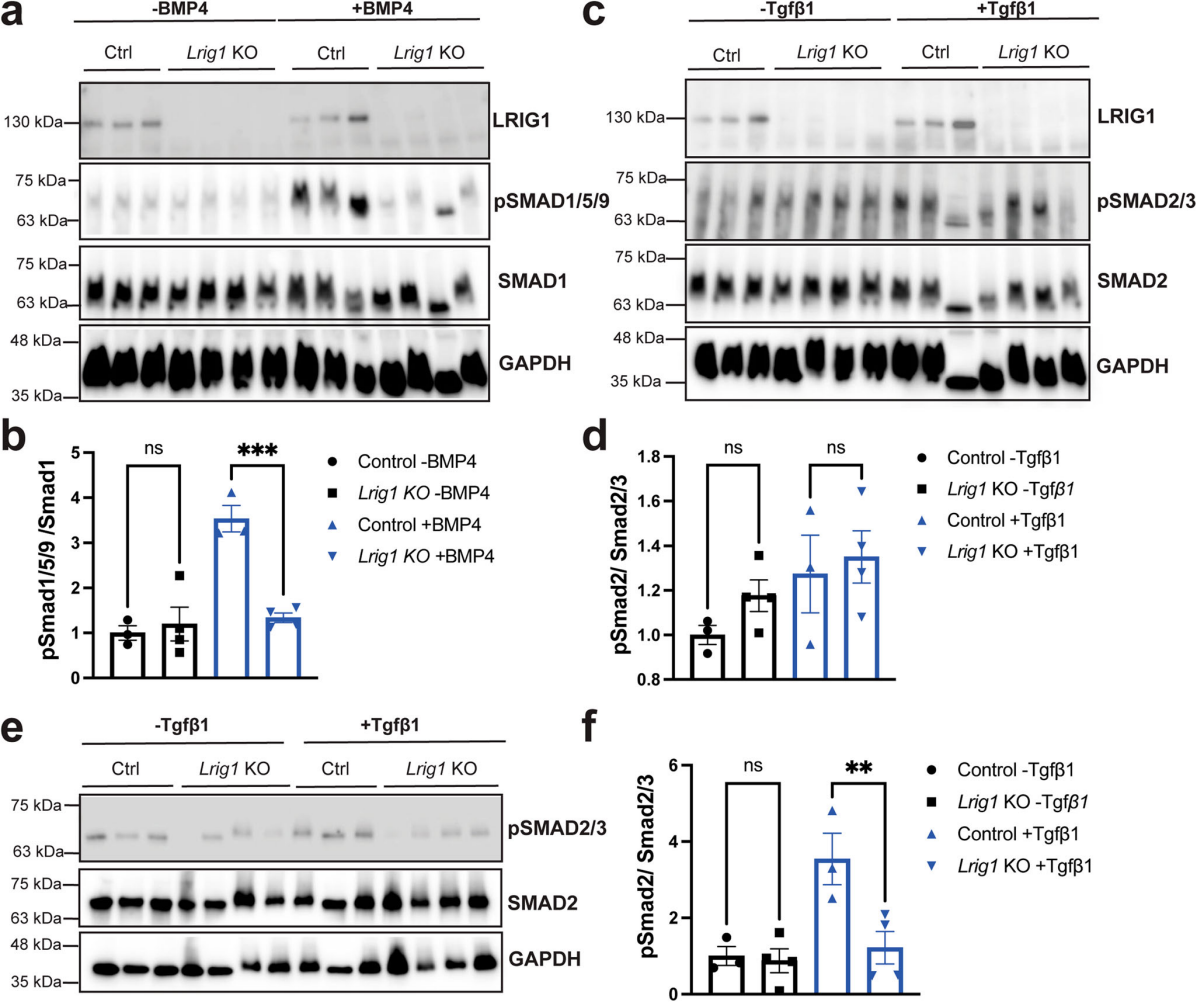
LRIG1 facilitates BMP and TGF*β* signaling at the cell surface by controlling Smad activation in a ligand-dependent manner. **a** Control (Ctrl) and *Lrig1* KO N2a clonal cell lines, serum starved overnight and then treated with (+) or without (-) BMP4 for 30 min at 37 °C and western blotted for LRIG1 and pSMAD1/5/9 stripped and re-probed for SMAD1 and GAPDH (loading control). See also Supplementary Fig. S3a. **b** Quantification of pSMAD1/5/9 levels relative to SMAD1 and normalized to the Ctrl -BMP4 condition. **c** Control (Ctrl) and *Lrig1* KO N2a clonal cell lines, serum starved overnight and then treated with (+) or without (-) TGF*β*1 for 30 min at 37 °C and western blotted for LRIG1 and pSMAD2 stripped and re-probed for SMAD2/3 and GAPDH (loading control). See also Supplementary Fig. S3b (**d**) Quantification of pSMAD2 levels relative to SMAD2/3 and normalized to the Ctrl - TGF*β*1 condition. **e** Control (Ctrl) and *Lrig1* KO N2a clonal cell lines transfected with both TGF*β*R1 and TGF*β*R2, serum starved overnight, and then treated with (+) or without (-) TGF*β*1 for 30 min at 37 °C and western blotted for LRIG1 and pSMAD2 stripped and re-probed for SMAD2/3 and GAPDH (loading control). See also Supplementary Fig. S3c (**f**) Quantification of pSMAD2 levels relative to SMAD2/3 and normalized to the Ctrl -TGF*β*1 condition. Source data for graphs are included in Supplementary Data 2.

If loss of LRIG1 impairs TGF*β* superfamily signalling pathways and thus facilitates these pathways under physiological conditions, we wondered how and where LRIG1 might be acting in these pathways to facilitate signalling, of which little is known. Thus, in a final series of experiments we made use of CRISPR-based approach to study these pathways in cultured cells. Here, we produced four control (Ctrl) and three *Lrig1* KO clonal N2a cell lines by transfecting with CRISPR/Cas9 plasmids and performing limiting dilution. Loss of LRIG1 was first confirmed using western blotting (Fig. 7a, c & Supplementary Fig. S3a, b). Next, we plated these cell lines, serum starved them overnight, and then treated them with or without BMP4 or TGF*β*1 at 10 ng/ml for 30 min. In the case of BMP4, we showed that treatment for 30 min robustly increased the amounts of pSMAD1/5/9 (relative to total SMAD1) as assessed by western blotting in the Ctrl cell lines demonstrating the activity of this pathway in these cell lines (Fig. 7a, b & Supplementary Fig. S3a). The levels of pSMAD1/5/9 in the *Lrig1* KO cell lines, however, were heavily impaired (Fig. 7b) suggesting that LRIG1 acts to facilitate BMP signalling via increasing pSMAD1/5/9. Interestingly, the impacts of LRIG1 on pSMAD1/5/9 phosphorylation only occurred in the context of ligand addition and not under homeostatic conditions, suggesting that LRIG1’s action is ligand-dependent.

In the case of TGF*β*1 treatment, we did not observe an increase in pSMAD2 levels (relative to total SMAD2/3) after 30 min of treatment in the Ctrl cell lines (Fig. 7c, d & Supplementary Fig. S3b), suggesting that TGF*β* signalling is normally operative at low levels in N2a cells, a finding that is further supported by the low expression of TGF*β*R2 we observed by western blotting (Supplementary Fig. S4c) and in published RNA-seq data from N2a cells^27^. Thus, to overcome the low levels of TGF*β* signalling in these cells, we co-expressed TGF*β*R1 and TGF*β*R2 in each of these Ctrl and *Lrig1* KO N2a cell lines, serum starved them overnight and treated them with and without 10 ng/ml TGF*β*1 for 30 min. As expected, overexpressing the TGF*β*-receptors in these cell lines lead to robust pathway activation as assessed by pSMAD2 in the Ctrl cell lines. Moreover, we observed significant impairment of pSMAD2 levels in the *Lrig1* KO cell lines that only occurs in the presence of ligand (Fig. 7e, f & Supplementary Fig. S3c) suggesting as above for BMP4 that LRIG1 can act to facilitate signalling via controlling SMAD phosphorylation at the cell surface in a ligand-dependent manner. Lastly, to assess whether facilitation of SMAD phosphorylation also occurs in a more physiologically relevant system we cultured secondary neurospheres from adult *Lrig1* KO and WT mice and then treated these spheres with BMP4 for 30 min as above. As was the case in N2a cells, we observed impairments in BMP4-induced SMAD1/5/9 phosphorylation in the context of loss of LRIG1 in these cells (Supplementary Fig. S5), revealing the importance of this pathway and function in adult neural stem cells.

## Discussion

The data we describe here, support three major conclusions. First, loss of LRIG1 in vivo results in increased proliferation of NSCs without impacting their neuronal progeny. Second, our data suggest that instead of acting through EGFR, LRIG1 likely acts via facilitation, broadly, of TGF*β* superfamily signalling pathways in cells found in the V-SVZ. Finally, and perhaps most importantly, we show that LRIG1 interacts directly with both TGF*β* and BMP receptors, the TGF*β*1 ligand, and leads to pathway facilitation by controlling SMAD phosphorylation at the cell surface. To the best of our knowledge this is the first time that LRIG1 has been shown to interact directly with the TGF*β* receptor, it’s ligand and the mechanism by which LRIG1 facilitates these pathways.

Previously, we showed that *Lrig1* expression increases in RGPs in the developing brain during a time when these cells transition into adult NSCs^14^. Analogously to the work described here, there we showed the loss of LRIG1 leads to enhanced proliferation of RGPs, however, in the developing brain we showed that enhanced proliferation occurs via increased activation of EGFR^14^. By extending these studies to adult NSCs in the V-SVZ, we wondered if LRIG1 would act similarly as it does in the developing brain, or would a different mechanism of action be in place given that LRIG1 and EGFR are more highly expressed at different stages of the lineage progression of adult NSCs. Thus, the work described here aimed to understand the role of LRIG1 in the proliferation of adult NSCs and the mechanistic details underlying its function. During the course of carrying out our studies, other groups published broadly consistent findings on LRIG1’s ability to control proliferation of adult NSCs^16,17^, and thus collectively provide strong consensus on the role of LRIG1 in the control of proliferation of these cells. We further extend these studies to show that there are no changes in the number of DCX-positive new-born neurons/neuroblasts in the V-SVZ or interneurons in the OB. However, the mechanisms underpinning the increased proliferation of adult NSCs occurs has remained unclear^28^. The work described here provides new details on how LRIG1 controls proliferation of adult NSCs.

We demonstrate that LRIG1 loss leads to hyperproliferation of adult NSCs by acting independently of EGFR signalling in vivo. Specifically, no differences in pEGFR immunostaining and immunoblotting were found between *Lrig1* KO and WT models in the adult V-SVZ. Bulk RNA-sequencing results from the adult V-SVZ of *Lrig1* KO mice showed differential expression of genes targeted by the canonical TGF*β* superfamily and led us to assess pathway activation in vivo with pSMAD staining. We show that loss of LRIG1 impairs both pSMAD2 and pSMAD1/5/9 driven signalling pathways. On the biochemical level, we show that LRIG1 is able to associate with components of both the BMP and TGF*β* branches of the TGF*β* superfamily and facilitate activity of their downstream signalling effectors. Further, we demonstrate both in vivo and in vitro, LRIG1 interacts with TGF*β*RI and TGF*β*RII, as well as with TGF*β*1 ligand. Finally, we show that in vitro, LRIG1 functions to facilitate TGF*β* and BMP signalling by controlling SMAD phosphorylation in a ligand-dependent manner. With respect to this latter point, we note the high expression of TGF*β*1 in microglia found in the V-SVZ and to a lesser extent BMP4 expressed in fibroblasts and mural cells suggesting locations from which these ligands may be derived in vivo. Further studies would be required to clarify the sources of these signals and their facilitation by LRIG1.

Proteins containing leucine rich repeat (LRR) are efficient in facilitating protein-protein interactions contributing to diverse regulatory roles throughout the brain and peripheral tissues^29^ Previous evidence has demonstrated that proteins rich in LRRs are able to bind to EGFR as well as TGF*β*Rs. As an example, Decorin, a small soluble leucine rich proteoglycan, has overlapping regions with LRRs in the LRIG1 ECD, and was found to facilitate signalling for EGFR and TGF*β*Rs^30–32^. Only a handful of studies have explored LRIG1’s interaction with receptors of the canonical TGF*β* superfamily. For example, Guimenny et al, used non-neuronal cell lines and *C.elegans* to discover both direct and genetic interactions between BMPR’s and LRIG1 via a series of immunoprecipitation assays^21^ and animal physiology. Additionally, Ferguson et al demonstrated dysregulation in BMP signalling as assessed by SMAD expression via immunostaining and western blotting in a *Lrig1* KO glioblastoma cell line and others have begun exploring the TGF*β* superfamily and LRIG1 in other cell types^33,34^.

Here, we present evidence that LRIG1 binds directly to TGF*β*1 in vitro and can promote signalling in the TGF*β* branch in adult neural stem cells. Other studies have also demonstrated the importance of LRR proteins such as LRRC33 and GARP, prior to activation of the mature TGF*β*1^35,36^. Based on the findings that LRIG1 can interact with multiple components of the TGF*β* family, it is possible LRIG1 could be acting as a coreceptor, promoting TGF*β* and BMP signalling. Other coreceptors, such as betaglycan (TGF*β*RIII), have been well characterized and are known to bind to several components of the TGF*β* branch such as TGF*β* type I and II receptors as well as to all three TGF*β* ligands^37^. Moreover, the dissociation constant that we found for LRIG1 and TGF*β*1 is similar to what is reported in the literature between betaglycan and all TGF*β* ligands^38^.

An important question that remains is, how LRIG1 functions to control SMAD phosphorylation. Our group (unpublished observation) and others have shown that LRIG1 is expressed on the membrane of early endosomes yet its functional role therein remains elusive^10^. Since TGF*β* and BMP signalling can occur both on the cell membrane and in early endosomes^39^ it is possible that LRIG1 could be promoting clathrin-mediated endocytosis following TGF*β* and BMP stimulation. Alternatively, cytoplasmic retention factors such as Smad Anchor for Receptor Activation (SARA), which is responsible for delivering SMADs for phosphorylation to TGF*β*RI^40^ could work in concert with LRIG1 wherein LRIG1 could promote recruitment of SARA. Whether LRIG1 recruits SARA, functions via endosomes or through some other unknown mechanism will require further study.

In terms of limitations of this study our data has two considerations. First, even though we did not find any support for aberrant activation of the EGFR pathway in the context of LRIG1 loss-of-function, we cannot rule out a minor contribution of the EGFR pathway in adult NSCs when LRIG1 is lost. Second, the knockout strategy used here is a constitutive loss-of-function and developmental confounds cannot be ruled out. Further studies using conditional knockout for *Lrig1* would be important future considerations.

In summary, our data reveals the loss of LRIG1 leads to increased proliferation of adult NSCs, without impacting neuronal progeny, and implicates canonical TGF*β* pathways as a mechanism by which LRIG1 regulates proliferation of these cells.

### Supplementary information

Supplementary Information

Description of Additional Supplementary Files

Supplementary Data 1

Supplementary Data 2

## Data Availability

Both raw and processed RNA-seq data are available at NCBI GEO under accession number: GSE263575 and code for these analyses are available at https://github.com/Yuzwalab/. Data shown in graphs are included in Supplementary Data 2.
