# Supplementary material for: LRIG1 controls proliferation of adult neural stem cells by facilitating TGFβ and BMP signalling pathways
Source: Commun Biol. 2024 Jul 10;7:845. doi: 10.1038/s42003-024-06524-8 (PMC11237139; doi:10.1038/s42003-024-06524-8)
Supplement: Supplementary file 1 — Supplementary Information [file 42003_2024_6524_MOESM1_ESM.pdf]

Supplementary Information

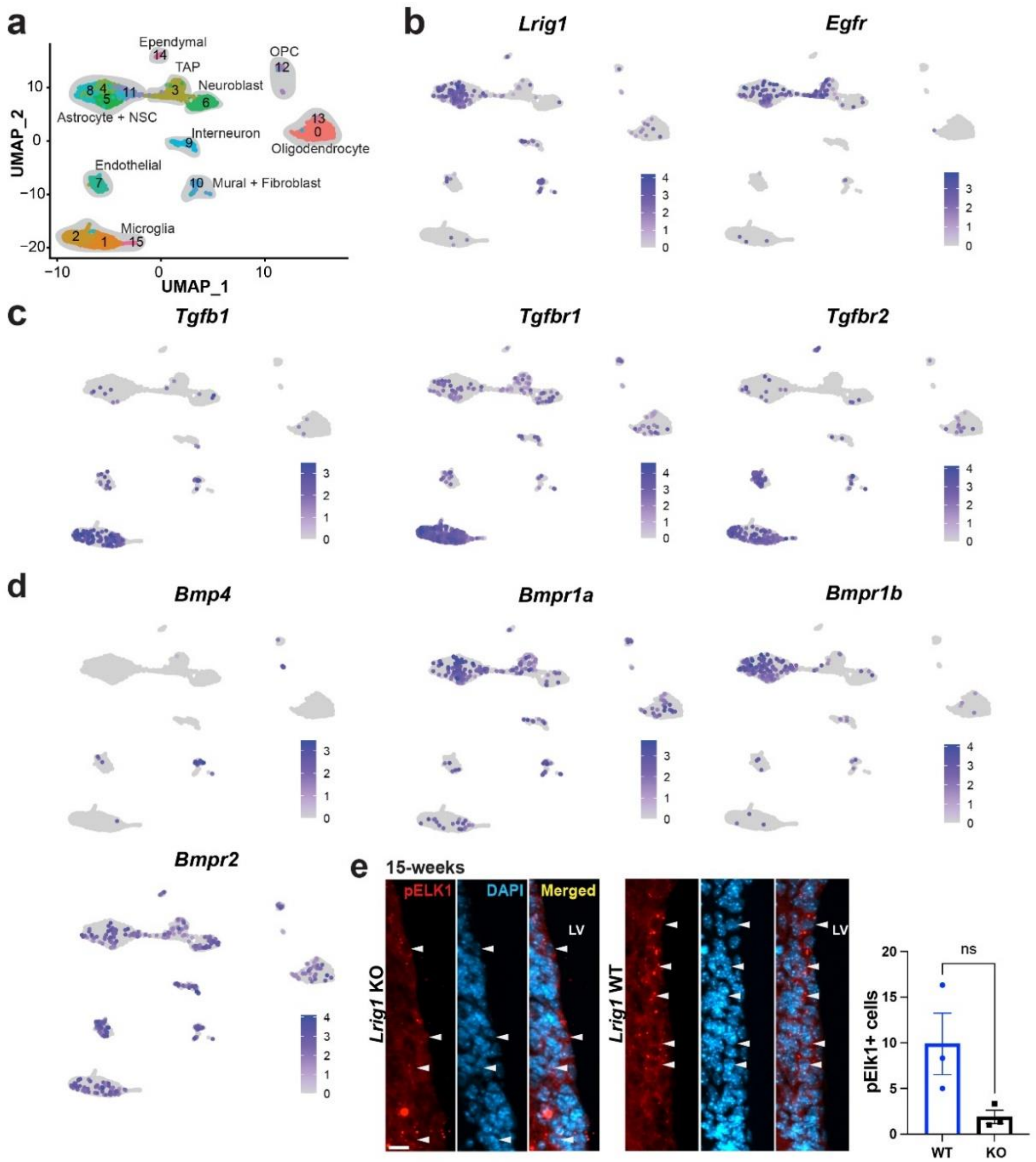

Supplementary Figure S1. Analysis of Mizrak et al. (GSE109447) scRNA-seq data from cells collected from the lateral wall V-SVZ area of 8-10 week old mice and pELK1 staining from *Lrig1* KO and WT mice. (**a**) Clustering and projection in two-dimensional space of scRNA-seq data in an uniform manifold approximation and projection (UMAP) plot. 14 clusters are identified and annotated as labelled on the plot. (**b**) Expression of *Lrig1* and *Egfr* overlayed on these scRNA-seq data shows that *Egfr* is more highly expressed in TAPs and activated NSCs. (**c,d**) Ligands and receptors for the TGFβ and BMP signalling pathways are expressed in cells found along the lateral wall as show by overlaying these genes on the scRNA-seq clusters from (A). In (**b-d**) the scale ranges from grey being the lowest to blue being the highest expression of the gene being analyzed. (**e**) Activated Elk1 (pELK1) staining along the lateral wall of the lateral ventricle of 15-week-old *Lrig1* and WT KO mice co-stained for Hoechst (left) and quantification of pELK1-positive cells along the lateral ventricle (right, N = 3 per group). Scale bar indicates 10 μm.

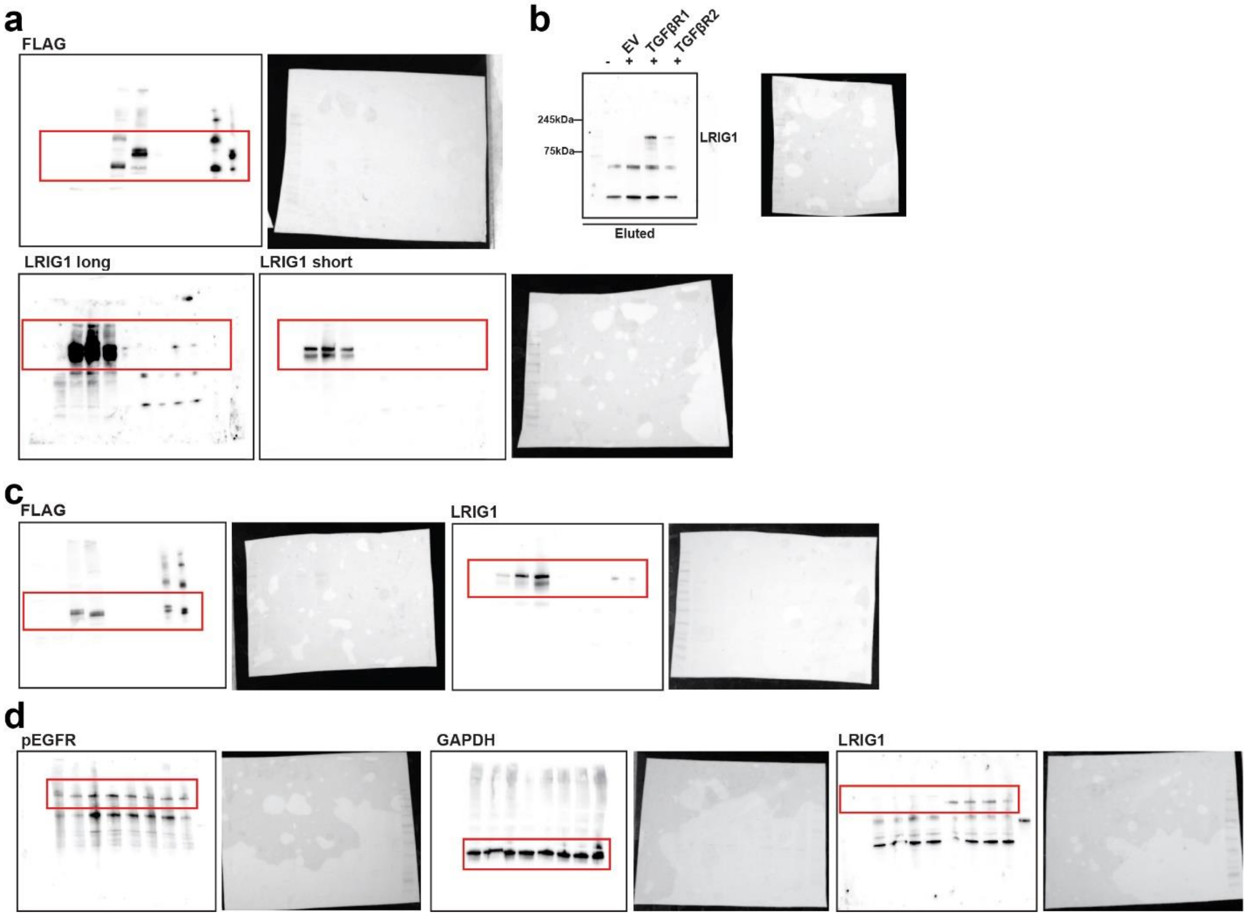

Supplementary Figure S2. Full western blot images for blots shown in Figures 3 and 6. (**a**) Blots shown in Figure 6a. (**b**) Eluted samples as shown in Figure 6a were re-run on a separate blot and probed for LRIG1 to enable band visualization without brighter bands associated with the load samples. (**c**) Blots shown in Figure 6b. (**d**) Blots shown in Figure 3c. In all panels, to the right of the blot is a white-light image of the blot membrane and red boxes indicate cropped areas shown in Figures 3 and 6.

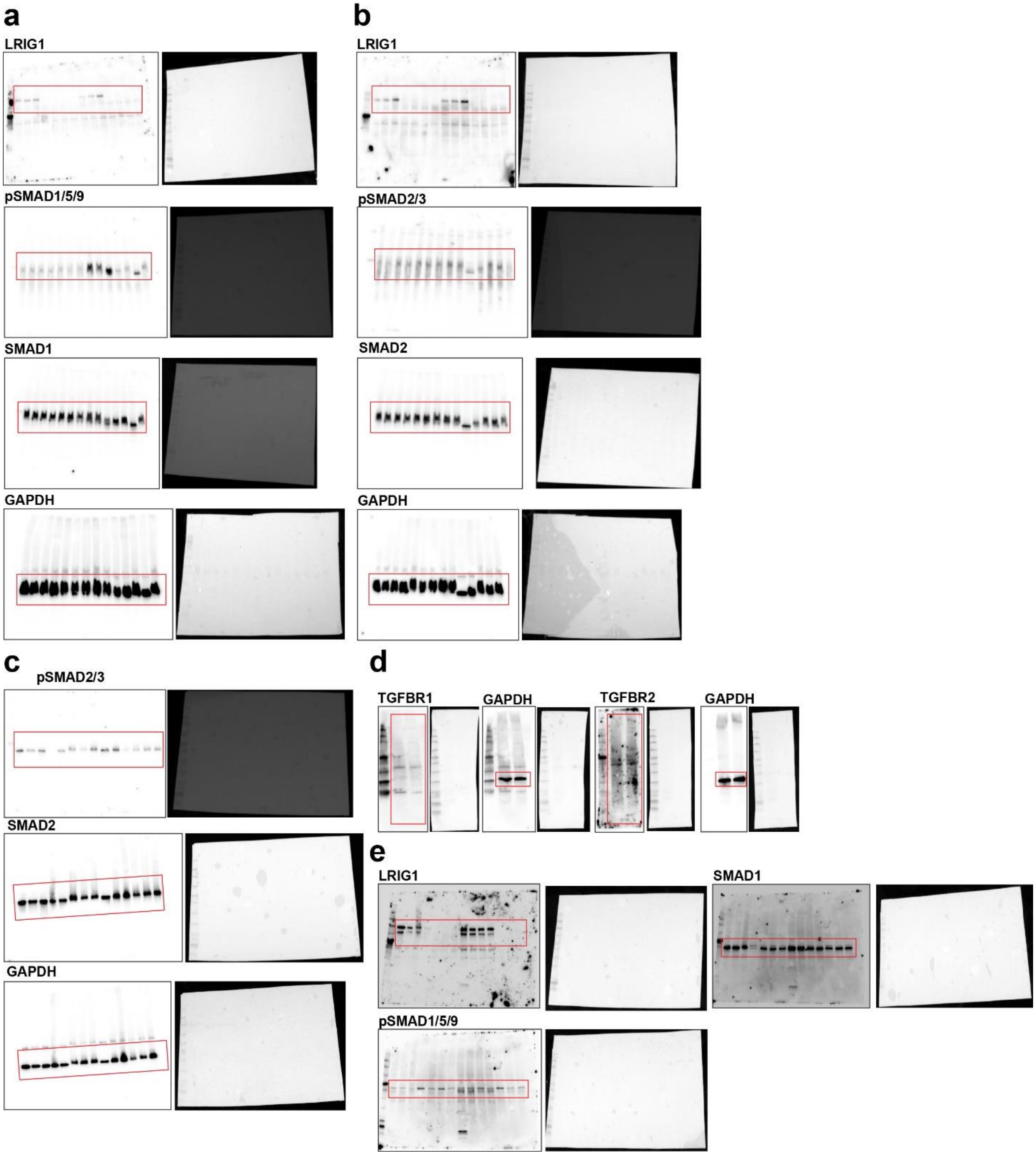

Supplementary Figure S3. Full western blot images for blots shown in Figure 7, Figure S4 and Figure S5. (**a**) Blots shown in Figure 7a. (**b**) Blots shown in Figure 7c (**c**) Blots shown in Figure 7e. (**d**) Blots shown in Supplementary Figure S4c. (**e**) Blots shown in Supplementary Figure S5. In all panels, to the right of the blot is a white-light image of the blot membrane and red boxes indicate cropped areas shown in Figure 7, Figure S4 and Figure S5.

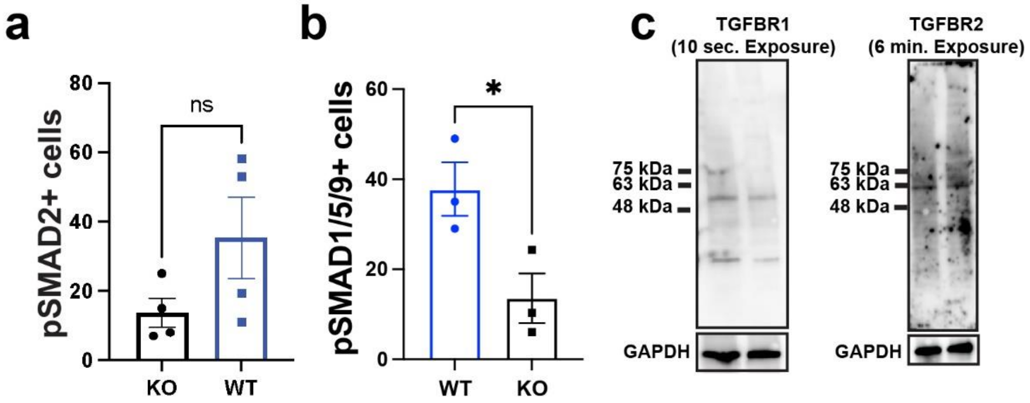

Supplementary Figure S4. Quantification of pSMAD2 or pSMAD1/5/9 positive cells in *Lrig1* KO and WT mice and expression of TGFβR1 and TGFβR2 in N2a cells by western blot.
(**a**) Quantification of the number of phosphorylated SMAD2 (pSMAD2) positive cells along the LW in 24-week old *Lrig1* KO and WT mice. N = 4 per group. (**b**) Quantification of the number of phosphorylated SMAD1/5/9 (pSMAD1/5/9) positive cells along the LW in 15-week-old *Lrig1* KO and WT mice. N = 3 per group. (**c**) Western blot using antibodies for TGFβR1 and TGFβR2 on wild-type N2a cells (both lanes) along with GAPDH as a loading control. TGFβR1 and TGFβR2 blots were probed and imaged at the same time. The exposure for TGFβR1 is 10 seconds while the exposure for TGFβR2 is 6 minutes supporting a substantial difference in expression of these two receptor subunits.

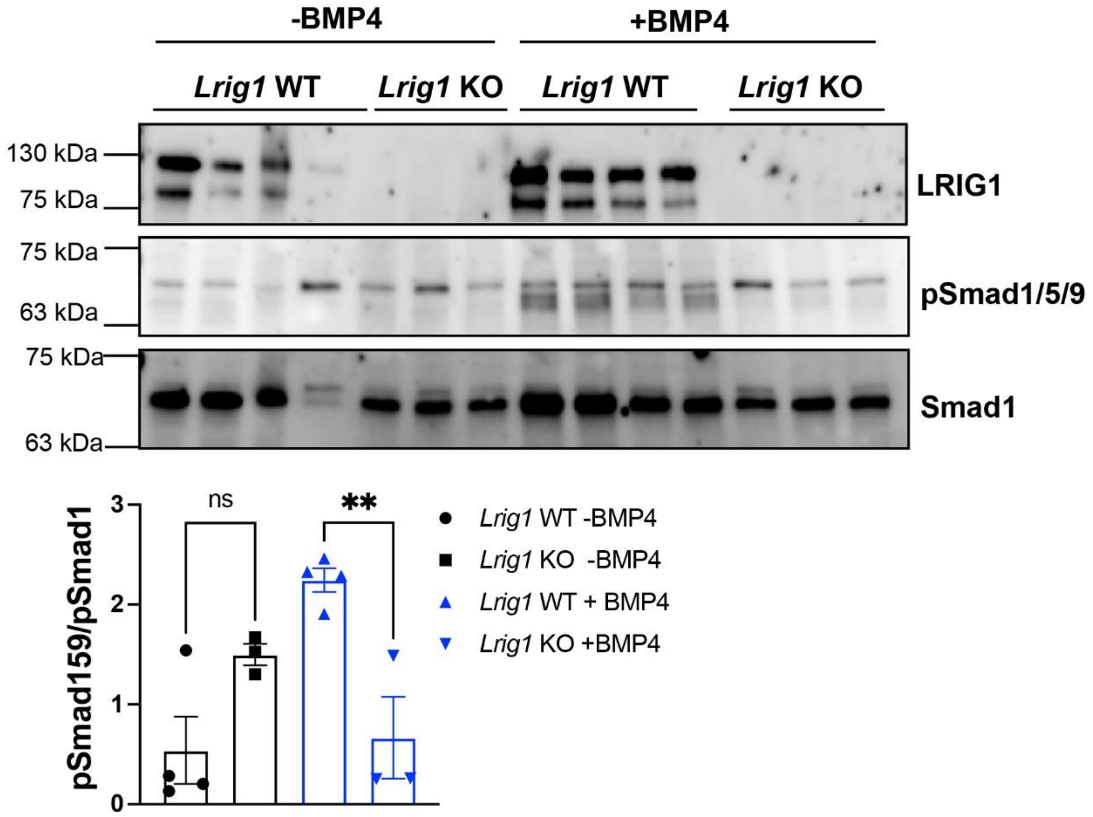

Supplementary Figure S5. LRIG1 facilitates BMP signaling in secondary neurospheres from 15-week old *Lrig1* KO and WT mice. Western blot for LRIG1 confirms the expected genotype and loss of LRIG1 and for pSMAD1/5/9 and Smad1 demonstrates impaired pSMAD1/5/9 levels in the context of BMP4 treatment of secondary neurospheres for 30 minutes (top) and quantification (bottom). N = 4 for WT neurospheres and N = 3 for KO neurospheres.

Supplementary Table S1: Primary antibodies used for western blot.

Antibody Name	Brand	Item/ID number	Dilution used
Mouse LRIG1 Antibody	R&D Systems	Cat #: AF3688, RRID:AB_2138836	1:500 (PLA 1:100)
Rabbit Anti-pEGFR	Abcam	Cat #: ab40815, RRID:AB_732110	1:1000
GAPDH (14C10) Rabbit mAb	Cell Signalling Technology	Cat #: 2118S, RRID:AB_561053	1:1000
DYKDDDDK Tag Rabbit Antibody	Cell Signalling Technology	Cat. #: 2368, RRID:AB_2217020	1:3000
Rabbit-anti-TGFbR1 Antibody (Alk5)	RayBiotech	Cat #:14464745	1:1000
Goat anti-Mouse TGF-beta RII Antibody	R&D Systems	Cat #: AF532, RRID:AB_355418	1:1000
Smad2/3 (D7G7) XP Rabbit mAb	Cell Signalling Technology	Cat #: 8685, RRID:AB_10889933	1:2000
SMAD1 (D59D7) XP Rabbit mAb	Cell Signalling Technology	Cat # 6944T, RRID:AB_10858882	1:2000
Phospho-Smad1 (Ser463/465)/Smad5 (Ser463/465)/Smad9(Ser465/467) (D5B10) Rabbit mAb	Cell Signalling Technology	Cat.#13820T	1:1000
Phospho-Smad2 (S465/467)(138D4) Rabbit mAb	Cell Signalling Technology	Cat#:3108	1:1000

Supplementary Table S2: Secondary antibodies used for western blots.

Antibody Name	Brand	Item/ID number	Dilution used
Peroxidase-conjugated AffiniPure Donkey Anti-Goat IgG (H+L)	Jackson Immuno Research Laboratories	Cat#: 705-035-003, RRID: AB_2340390	1:5000
Peroxidase-conjugated AffiniPure Goat Anti-Rabbit IgG (H+L)	Jackson Immuno Research Laboratories	Cat#: 111-035-003, RRID: AB_2313567	1:5000
Anti-mouse IgG, HRP-linked Antibody	Cell Signalling Technology	Cat#: 7076S, RRID:AB_330924	1:5000
Streptavidin-HRP	Cell Signalling Technology	Cat#: 3999S, RRID:AB_10830897	1:5000

Supplementary Table S3: Primary Antibodies for IHC

Antibody Name	Brand	Item/ID number	Dilution used
Phospho-Smad2 (S465/467)(138D4) Rabbit mAb	Cell Signalling Technology	Cat. #3108	1:200
Phospho-Smad1 (Ser463/465)/Smad5 (Ser463/465)/Smad9(Ser465/467) (D5B10)Rabbit mAb	Cell Signalling Technology	Cat.#13820T	1:400
Mouse Anti-Ki67	BD Biosciences	Cat# 550609, RRID:AB_393778	1:50
Rabbit Anti-pEGFR	Abcam	RRID:AB_732110	1:250
Rabbit Anti-SOX2	Cell Signaling Technologies	Cat# 3728, RRID:AB_2194037	1:250
Chicken Anti-GFAP	EMD Millipore	Cat# AB5541, RRID:AB_177521	1:400
Mouse Anti-GFAP	Cell Signaling Technologies	Cat# 3670, RRID:AB_561049	1:300
Rabbit Anti-Calbindin	Cell Signaling Technologies	Cat# 13176, RRID:AB_2687400	1:200
Goat Anti-Calretinin	R&D Systems	Cat# AF5065, RRID:AB_2068516	1:200
Rabbit Anti-Doublecortin (DCX)	Cell Signaling Technologies	Cat# 4604, RRID:AB_561007	1:400
Mouse anti-phosphoElk1	Santa Cruz Biotechnology	Cat# sc-8406 RRID:AB_627509	1:100
Mouse anti-BMPR-IB/ALK-6 Antibody	R&D Systems	Cat# MAB505-100 RRID:AB_2227942	1:100 (PLA)

Supplementary Table S4: Secondary Antibodies for IHC and PLA

Antibody Name	Brand	Item/ID number	Dilution used
Alexa Fluor 488-Conjugated anti-Chicken Secondary Antibody	Jackson ImmunoResearch Laboratories	Cat# 703-545-155, RRID:AB_2340375	1:500
Alexa Fluor 647-Conjugated anti-Rabbit Secondary Antibody	Jackson ImmunoResearch Laboratories	Cat# 711-605-152, RRID:AB_2492288	1:500
Cy3-Conjugated anti-Mouse Secondary Antibody	Jackson ImmunoResearch Laboratories	Cat# 715-165-150, RRID:AB_2340813	1:500
Alexa Fluor 488-Conjugated anti-Goat Antibody	Jackson ImmunoResearch Laboratories	Cat# 705-545-147, RRID: AB_2336933	1:500
Duolink In Situ PLA Probe Anti-Mouse MINUS Affinity purified Donkey anti-mouse IgG (H+L)	Sigma-Aldrich	Cat# DUO92004-40TST, RRID:AB_2713942	1:10
Duolink In Situ PLA Probe Anti Goat PLUS	Sigma-Adrich	Cat# DUO92003-30RXN	1:10

Supplementary Table S5: Plasmids used for transfection experiments

Vector Name	Brand	Item/ID number
Mouse BMPRIB Gene ORF cDNA clone expression plasmid, C-Flag tag	Sino Biological	Cat#: MG50004-CF
Mouse TGFβR2 Gene ORF cDNA clone expression plasmid, C-Flag tag	Sino Biological	Cat#: MG57076-CF
Mouse TGFβR1/Alk5- Gene ORF cDNA clone expression plasmid, C-Flag tag	Sino Biological	Cat#: MG50238-CF
LRIG1 cDNA ORF Clone, Mouse, C-Myc tag	Sino Biological	Cat #: MG50511-CM
