# Supplementary material for: LRIG1 controls proliferation of adult neural stem cells by facilitating TGFβ and BMP signalling pathways
Source: Commun Biol. 2024 Jul 10;7:845. doi: 10.1038/s42003-024-06524-8 (PMC11237139; doi:10.1038/s42003-024-06524-8)
Supplement: Supplementary file 2 — Description of Additional Supplementary Files [file 42003_2024_6524_MOESM2_ESM.pdf]

Description of Additional Supplementary Files

File name: Supplementary Data 1
Description: Table of differentially expressed genes comparing WT to Lrig1 KO mice with adjusted p-value < 0.1.

File name: Supplementary Data 2
Description: The source data behind the graphs in the paper.
